# microRNA-2110 functions as an onco-suppressor in neuroblastoma by directly targeting *Tsukushi*

**DOI:** 10.1371/journal.pone.0208777

**Published:** 2018-12-14

**Authors:** Zhenze Zhao, Veronica Partridge, Michaela Sousares, Spencer D. Shelton, Cory L. Holland, Alexander Pertsemlidis, Liqin Du

**Affiliations:** 1 Department of Chemistry and Biochemistry, Texas State University, San Marcos, Texas, United States of America; 2 Greehey Children’s Cancer Research Institute, UT Health San Antonio, San Antonio, Texas, United States of America; 3 Department of Cell Systems and Anatomy, The University of Texas Health, San Antonio, Texas, United States of America; 4 Department of Pediatrics, The University of Texas Health, San Antonio, Texas, United States of America; Universitat des Saarlandes, GERMANY

## Abstract

microRNA-2110 (miR-2110) was previously identified as inducing neurite outgrowth in a neuroblastoma cell lines BE(2)-C, suggesting its differentiation-inducing and oncosuppressive function in neuroblastoma. In this study, we demonstrated that synthetic miR-2110 mimic had a generic effect on reducing cell survival in neuroblastoma cell lines with distinct genetic backgrounds, although the induction of cell differentiation traits varied between cell lines. In investigating the mechanisms underlying such functions of miR-2110, we identified that among its predicted target genes down-regulated by miR-2110, knockdown of *Tsukushi (TSKU)* expression showed the most potent effect in inducing cell differentiation and reducing cell survival, suggesting that TSKU protein plays a key role in mediating the functions of miR-2110. In investigating the clinical relevance of miR-2110 and *TSKU* expression in neuroblastoma patients, we found that low tumor miR-2110 levels were significantly correlated with high tumor *TSKU* mRNA levels, and that both low miR-2110 and high *TSKU* mRNA levels were significantly correlated with poor patient survival. These findings altogether support the oncosuppressive function of miR-2110 and suggest an important role for miR-2110 and its target *TSKU* in neuroblastoma tumorigenesis and in determining patient prognosis.

## Introduction

Neuroblastoma is one of the most aggressive types of childhood cancers, accounting for ~15% of cancer-related childhood deaths [1, 2]. Studies have revealed that neuroblastoma was originated from neural crest precursor cells failing to complete the cell differentiation process [2, 3]. With the repression of the differentiation pathways, the precursor cells leave the normal differentiation process and adopt uncontrolled cell proliferation cycle at an undifferentiated state [4]. Due to this mechanism of tumorigenesis, inducing cell differentiation has been one of the key strategies to treat neuroblastoma. Only one differentiation agent, 13-retinoic acid (RA), has been proven to be successful to prevent the recurrence a subset of high-risk neuroblastomas [5, 6]. However, lack of response to RA treatment was found to be common in high-risk neuroblastoma patients [6]. Identification of new classes of differentiation agents, mechanistically different from RA, is still in demand for treating neuroblastoma resistant to RA.

In recently years, increasing number of genes, including protein-coding genes and genes for non-coding RNAs, involved in regulating neuroblastoma cell differentiation have been discovered, providing more and more diverse molecular targets for exploring new pathways to develop novel differentiation agents [7–12]. microRNAs (miRNAs), a class of small non-coding RNAs, haven been demonstrated to play a critical role in regulating neuroblastoma cell differentiation [12–16]. Due to the small size of miRNAs, their intracellular levels can be easily manipulated using synthetic oligonucleotides (oligos) [17], which make them stand out as one of the most prominent classes of therapeutic targets for developing a new class of differentiation therapy.

Previously, our group conducted a high-content screen (HCS) to systematically identify candidate miRNAs that function as inducers of neuroblastoma cell differentiation, by applying a library of microRNA mimics, synthetic oligos developed to mimic the function of endogenously expressed microRNAs [12]. Through the screen, we identified a group of miRNA mimics that potently induce neurite outgrowth, the morphological differentiation marker of neuroblastoma cells [12, 14, 18]. miR-2110 was among the group of neurite-inducing miRNAs [12], suggesting its differentiation-inducing and oncosuppressive function. However, such function and the underlying mechanisms have not been further characterized. In the current study, we investigated the function of miR-2110 in regulating neuroblastoma cell survival and differentiation in multiple neuroblastoma cell lines, and investigated the target genes that mediate such functions of miR-2110. We also evaluated the potential clinical relevance of the miR-2110-mediated regulatory pathway in neuroblastoma patient prognosis.

## Materials and methods

### Reagents and cell lines

miR-2110 mimic, siRNAs and negative control oligos were purchased from Dharmacon. Rabbit anti-βIII-tubulin, anti-GAP43, anti-NSE, anti-TSKU, anti-calnexin, and HRP-conjugated anti-rabbit IgG antibodies were obtained from Thermo Fisher Scientific. Anti-cleaved PARP was purchased from Cell Signaling Technology. BE(2)-C, SKNDZ, CHLA-90, and SKNFI cells were from the American Type Culture Collection (ATCC). Kelly cells were obtained from the cell line repository at the Greehey Children’s Cancer Research Institute at the University of Texas Health San Antonio. Cells were grown in DMEM/F12 (Corning Cellgro) supplemented with 10% Equafetal bovine serum (Atlas Biologicals).

### Detection of neurite outgrowth and cell proliferation

2,500 cells were plated and treated in 96-well plates. For measuring neurite outgrowth, cells were placed in an IncuCyte ZOOM Live Cell Imaging System (Essen BioScience), with cell images taken under 20X magnification every 12 hours for 4 days. Neurite lengths and cell confluence were calculated as described previously [12]. Cell proliferation was determined by measuring increase in cell confluence over time.

### Western blots

Cell lysates were prepared using 25 mM Tris-HCl buffer (pH 7.4) with 1% Triton X-100. Protein concentration was determined by BCA assay (Pierce). Equal amounts of cell lysates were loaded into 10% SDS-PAGE to separate proteins by size. Proteins were transferred to PVDF membranes (Thermo Fisher Scientific) for detecting specific proteins with antibodies. Blots were visualized using SuperSignal West Pico Chemiluminescent Substrate (Thermo Fisher Scientific) on the Molecular Imager ChemiDoc XRS+ (Bio-Rad Laboratories).

### Cell viability assay

Cell viability was measured by MTT (2-(4,5-Dimethylthiazol-2-yl)-2.5-Diphenyltetrazolium Bromide) assay. Briefly, cells were plated in 96-well plates and treated as specified. MTT (15 μL at 2.5 mg/mL in 1X PBS) was then added and incubated for 1 hour at 37°C. Precipitates were spun down and dissolved in DMSO. Specific absorbance at 570 nm were measured to determine relative cell viability.

### Colony formation assay

2,000 cells were transfected with the specified oligos at 25 nM and plated into 10 cm dishes. Two weeks later, cell colonies were stained with 0.5% crystal violet. The number and size of colonies were quantified with Image J (NIH).

### Caspase 3/7 activity assay

2,500 cells were transfected with the specified oligos at 25 nM and plated into 96-wells plates. Five days later, caspase 3/7 activity was measured using caspase 3/7 activity assay kit (Promega Inc).

### mRNA expression array

Total RNA was isolated using the mirVana miRNA isolation kit (Life Technologies). mRNA expression profiling was performed using the Illumina mRNA WG-6 v3 microarray platform.

### Luciferase reporter assay

DNA fragments (900bp) of the wild-type (WT) 3’UTR containing all three predicted target sites of miR-2110 were amplified from human genomic DNA. The amplified WT 3’UTR was inserted downstream of firefly luciferase coding sequences into pmirGLO dual-luciferase reporter (Promega), a vector that contains both firefly and Renilla luciferase cDNAs under the control of separate promoter/terminator systems, to generate the TSKU-WT reporter. Three mutant reporters with mutations of the seed sequences at each predicted target site was generated (TSKU-mut1, TSKU-mut2 and TSKU-mut3) using site-directed mutagenesis kit. A mutant reporter with all three predicted target sites mutated was also generated. BE(2)-C cells were co-transfected with the specified luciferase reporters (0.8 ng/μl) and oligos (miR-2110 mimic or control oligo, 25 nM). Luciferase activities were measured after 72 hours and the effect of miR-2110 was determined as previously described [14].

### Statistical survival analysis of neuroblastoma patients

Survival analyses were based on three published datasets, the SEQC neuroblastoma patient dataset which includes 498 patients, the NRC neuroblastoma dataset which includes 283 patients with primary untreated neuroblastoma tumors, and the neuroblastoma TARGET (Asgharzadeh) dataset which includes 249 patients [19] (http://r2.amc.nl). To evaluate the correlation of neuroblastoma tumor miR-2110 or TSKU mRNA levels with patient survival in each dataset, the neuroblatoma patients were divided into two groups based on miR-2110 or TSKU mRNA levels. The statistical significance of the difference in miR-2110 or TSKU mRNA levels between the two patient groups were examined by unpaired t-test, with *p* < 0.05 considered as statistically significant. Patient survival analyses were performed using the Kaplan-Meier method, and statistical significance of difference between groups was determined by 2-tailed log-rank test with *p* < 0.05 considered statistically significant.

### Other statistical analysis

To evaluate the effect of the panel of 40 siRNAs on neurite outgrowth, the *p*-value for neurite length associated with each siRNA was determined by comparing to the mean neurite length of the whole panel using multiple-sample *t-*test. An increase in neurite length with *p* < 0.05 and False Discovery Rate (FDR) < 0.2 was considered as statistically significant. For all other experiments, the statistical significance for each treatment was determined by two-tailed Student’s *t-*test by comparing the treatment group with the control group, with *p* < 0.05 considered statistically significant.

## Results

### miR-2110 over-expression induces cell differentiation and inhibits cell survival and proliferation in BE(2)-C cells

**Fig 1:**
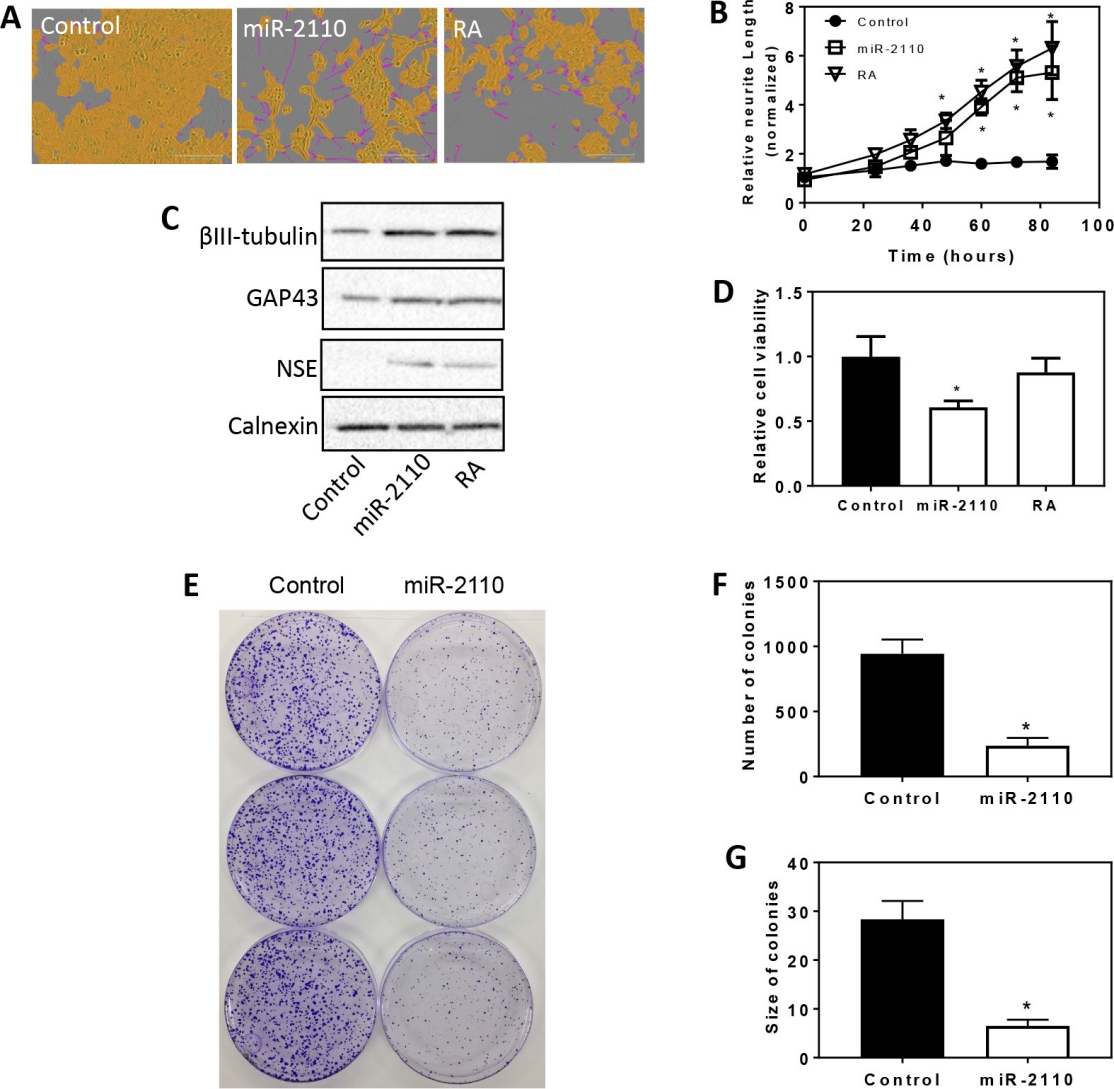
Effect of miR-2110 overexpression on BE(2)-C cell differentiation, survival and proliferation. **(A-B),** 2,500 cells were transfected with control oligo (25 nM), miR-2110 mimic (25 nM) or treated with RA (0.5 μM) in triplicate in 96-well plates. Neurite outgrowth was measured every 12 hours for 4 days. **A,** Representative cell images analyzed to define neurites (pink) and cell body areas (yellow) after 4 days of transfection. **B**, Time-dependent effect of miR-2110 mimic and RA on neurite outgrowth. *, *p* < 0.05 compared to control at the respective time points. **C**, Effect of miR-2110 mimic and RA treatment on protein expression levels of cell differentiation markers. Cells were transfected or treated with the indicated molecules at above. After 4 days, protein lysates were collected and differentiation markers βIII-tubulin, GAP43 and NSE were determined by Western blot with calnexin protein level as a loading control. **D,** Effect of miR-2110 mimic and RA on cell viability. Cells were transfected as above, and cell viability was measured after 4 days. *, *p* < 0.05 compared to control. (**E-G**), Effect of miR-2110 mimic and RA on colony formation of BE(2)-C cells. Shown are plate images (**E**) and quantification of the number (**F**) and size (**G**) of colonies. *, *p* < 0.05 compared to control.

We previously identified miR-2110 mimic as inducing neurite outgrowth in a neuroblastoma cell line BE(2)-C by HCS [12], suggesting that miR-2110 functions as an inducer of cell differentiation. However, additional cell differentiation traits were not investigated. Here we further investigated the effect of miR-2110 overexpression on expression of molecular differentiation markers and on cell survival in BE(2)-C, a cell line previously shown to be sensitive to differentiation induction by RA and differentiation-inducing miRNAs [12]. As shown in **Fig 1A and 1B,** treatment of BE(2)-C cells with miR-2110 mimic (25 nM) significantly induced neurite outgrowth, with its effect comparable to RA (0.5 μM), a differentiation agent currently used to treat high-risk neuroblastoma, recapitulating our previous findings [12]. As shown in **Fig 1C**, miR-2110 mimic dramatically increased protein expression levels of cell differentiation markers βIII-tubulin, growth associated protein 43 (GAP43), and neuron specific enolase (NSE) relative to control, and the extent of over-expression was comparable to what was observed with RA treatment. Remarkably, miR-2110 mimic showed more dramatic effect in decreasing cell viability than RA (**Fig 1D**). Moreover, as shown in **Fig 1E–1G**, miR-2110 mimic reduced colony forming capacity, reducing both colony size and number, of BE(2)-C cells relative to controls. Together, these results demonstrate that miR-2110 is an inducer of BE(2)-C cell differentiation and that the induced differentiation is coupled with reduced cell survival and proliferation.

### *Tsukushi (TSKU)* is identified as the most potent regulator of BE(2)-C cell differentiation and survival among the predicted direct target genes of miR-2110

In order to identify the direct target genes of miR-2110 that mediate its differentiation-inducing and cell survival-reducing function, we used Ingenuity Pathway Analysis (IPA, Qiagen) to identify the predicted targets of miR-2110. We then exploited expression microarrays to determine which of the predicted genes are down-regulated at mRNA levels by miR-2110 mimic in BE(2)-C cells. These combined analyses identified 21 predicted targets that were down-regulated by ≥40% at the mRNA level by miR-2110 mimic (**Table 1**). The entire gene expression array dataset used to generate Table 1 is shown in **S4 Table**.

**Table 1:** The 21 predicted target genes of miR-2110 that are down-regulated at the mRNA level by ≥40% by miR-2110 mimic.

(a) Gene name	(b) Ratio of mRNA level
	(miR-2110 vs. control)
USP13	0.45
SYN1	0.47
FOXM1	0.48
TSKU	0.48
G3BP1	0.50
RALY	0.51
SLC25A23	0.51
E2F2	0.53
PATL1	0.53
NOP56	0.54
STRN4	0.54
PMM2	0.55
SLC35E1	0.56
DDN	0.57
TMEM69	0.57
MARCKSL1	0.58
TTL	0.58
ELK1	0.59
SLC38A1	0.59
OAF	0.60
SORBS3	0.60

Shown are (a) the gene name and (b) the ratio of mRNA levels in cells treated with miR-2110 mimic to cells treated with control oligo (25 nM).

**Fig 2:**
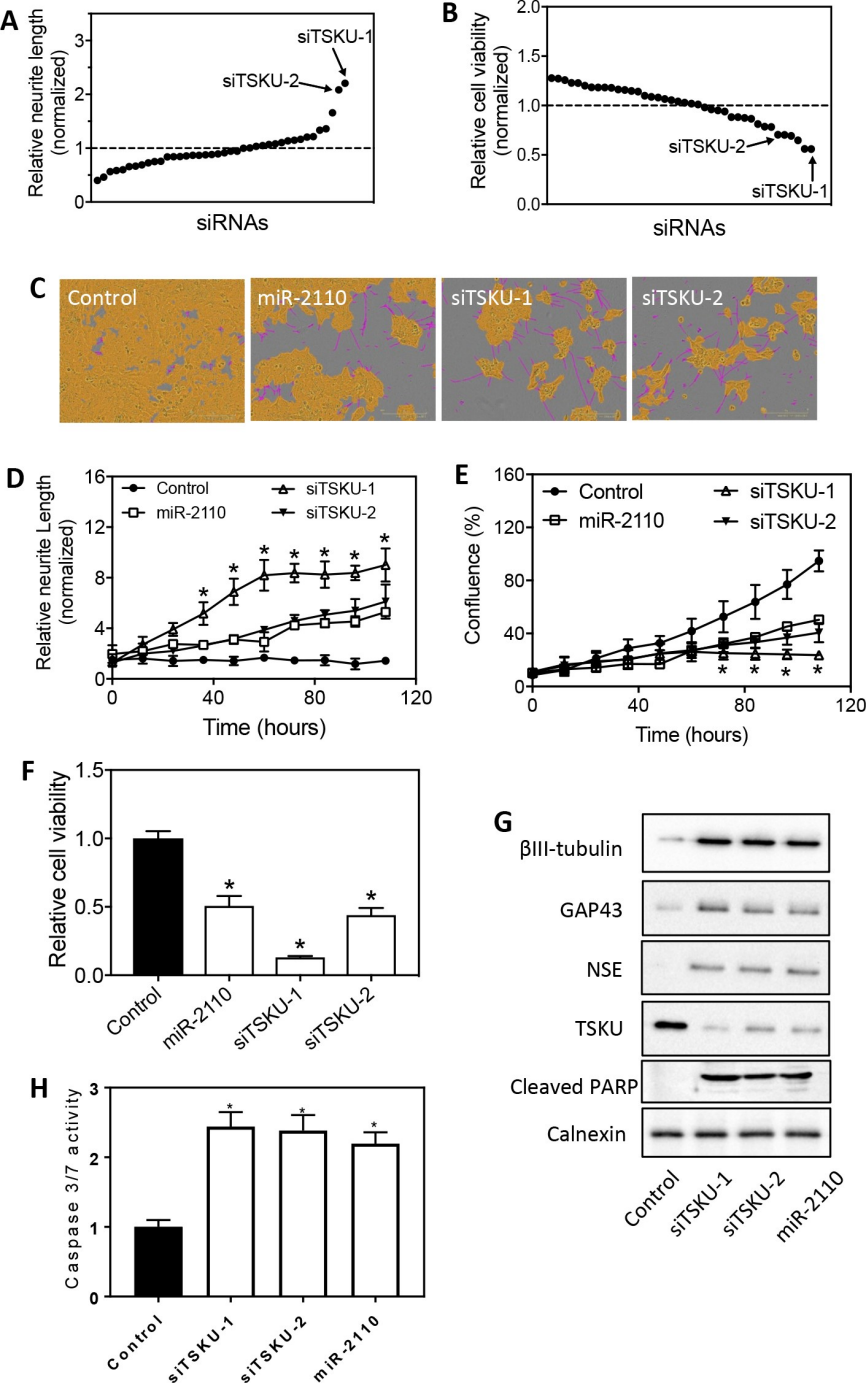
Identification of TSKU as miR-2110 target that regulates BE(2)-C cell differentiation. (**A-B**) HCS of siRNAs against the 21 predicted target genes of miR-2110 identifies siRNAs that induce neurite outgrowth and reduce cell viability in BE(2)-C cells. Cells were transfected with each siRNA (25 nM) in triplicate and cultured for 4 days. Neurite outgrowth and cell viability was measured as above. **A,** Scatter plot of normalized neurite lengths, sorted in ascending order, associated with individual siRNAs. **B,** Scatter plot of normalized cell viabilities, sorted in descending order, associated with individual siRNAs. Shown are the mean values of the normalized neurite lengths and cell viabilities derived from three independent experiments. Dotted lines are the means of the library. (**C-F**) Effect of siTSKUs on neurite outgrowth and cell viability was validated in separate experiments using re-ordered siTSKUs. Cells were transfected with the indicated oligos (25 nM) and neurite outgrowth and cell confluence were measured every 12 hours for 4 days. Cell viability was measured at the end-point of the experiment. **C**, Representative cell images analyzed to define neurites and cell body areas after 4 days of treatment. (**D-E**), Time-dependent neurite outgrowth (**D**) and cell confluence change (**E**) under each treatment. *, *p* < 0.05 for all three treatments (miR-2110, siTSKU-1 and siTSKU-2) compared to the control at the respective time points. **F,** Cell viability associated with each treatment. *, *p* < 0.05 compared to control. (**G)** Effect of siTSKUs and miR-2110 mimic on protein levels of cleaved PARP and cell differentiation markers. Cells were transfected and protein levels were measured as above. (**H)** Effect of siTSKUs and miR-2110 mimic on caspase 3/7 activity. *, *p* < 0.05 compared to control.

To examine whether the 21 genes play a role in regulating neuroblastoma cell differentiation and survival, we first examined whether knockdown of each gene expression by siRNA induces neurite outgrowth and reduces cell survival in BE(2)-C cells. At least two siRNAs targeting different sites for each mRNA were used. As shown in **Fig 2A** and **S1 Table**, five siRNAs were identified to significantly induce neurite outgrowth, among which the two siRNAs against *TSKU* (siTSKU) showed the most potent effect. Further examination of cell survival showed that the two siTSKUs were also among the top siRNAs that significantly reduced cell survival (**Fig 2B** and **S2 Table**), whereas the other three neurite-inducing siRNAs (siE2F2, siFOXM1 and siNOP56-1) did not significantly reduce cell viability. Together, these results suggest that TSKU plays the major role in mediating the differentiation-inducing function of miR-2110 in BE(2)-C cells. Images in **Fig 2C** indicate BE(2)-cells treated with miR-2110 mimic and siTSKUs induce dramatic neurite outgrowth comparing to control. **Fig 2D and 2E** further shows that siTSKUs and miR-2110 mimic induced neurite outgrowth and reduced cell proliferation in a time-dependent manner. Consistent with the above results, miR-2110 mimic and siTSKUs significantly reduced cell survival (**Fig 2F**). In addition, siTSKUs increased expression of cell differentiation markers (**Fig 2G**), further demonstrating that knockdown of *TSKU* expression truly induced BE(2)-C cell differentiation. Protein levels of TSKU were also measured, confirming that TSKU protein expression was truly depleted by siTSKUs (**Fig 2G**). Previous studies have shown that cell apoptosis was induced following neuroblastoma cell differentiation treated with differentiation agents [20]. We therefore examined whether miR-2110 mimic and siTSKUs induce apoptosis in BE(2)-C. As expected, miR-2110 mimic and siTSKUs increased both the cleaved PARP protein levels (**Fig 2G**) and the caspase 3/7 activity (**Fig 2H**), indicating cell apoptosis was truly induced by miR-2110 mimic and siTSKUs.

### The three predicted target sites of miR-2110 in the 3’UTR of *TSKU* mRNA are validated in BE(2)-C cells

**Fig 3:**
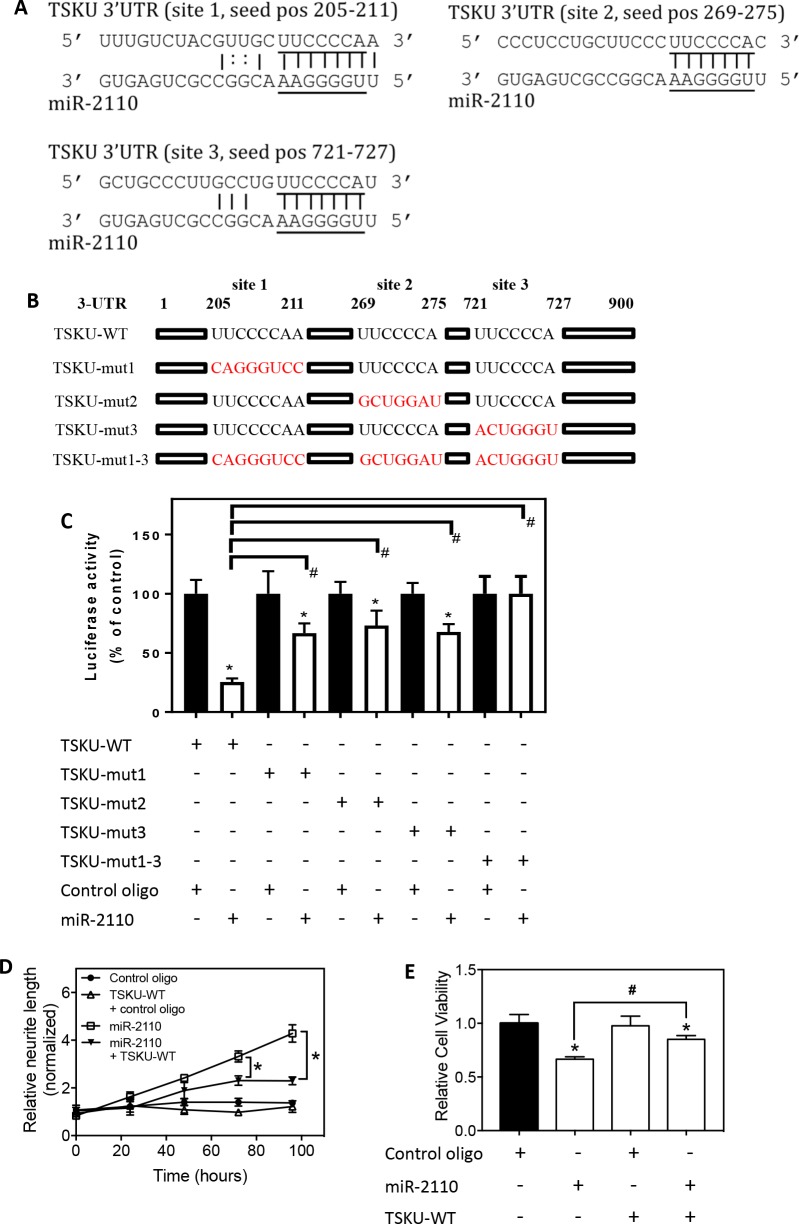
Validation of TSKU as direct target of miR-2110. (**A**) The predicted interactions between miR-2110 and its three target sites in the 3’UTR of TSKU mRNA. The seed sequences are underlined. pos, position. (**B**) The schematic diagram showing the mutations on predicted target sites of TSKU 3’UTR in the corresponding luciferase reporters. (**C**) Validation of the target sites by luciferase assay. BE(2)-C cells were co-transfected with the indicated vectors and oligos (miR-2110 mimic or control oligo). Three days after transfection, cells were lysed and luciferase activity was measured and analyzed for the indicated comparison. *, *p* < 0.05, comparing to cells co-transfected with the corresponding luciferase reporter and control oligo. #, *p* < 0.05 comparing each of the mutants (TSKU-mut1, TSKU-mut2, TSKU-mut3 and TSKU-mut1-3) to TSKU-WT. (**D-E**) Effect of TSKU 3’UTR over-expression on the function of miR-2110 mimic in inducing neurite outgrowth and in reducing cell viability. BE(2)-C cells were co-transfected with vectors (TSKU-WT or the control vector without 3’UTR of TSKU) and oligos (miR-2110 mimic or control oligo). Neurite outgrowth and cell viability were measured as above. **D**, Neurite outgrowth under each treatment. *, *p* < 0.05 comparing the indicated two treatments at the indicated time points. **E**, Cell viability under each treatment. *, *p* < 0.05 comparing to the corresponding control oligo-treated groups. #, *p* < 0.05 comparing the indicated two groups.

As shown in **Fig 3A**, the 3’UTR of *TSKU* mRNA contains three predicted target sites for miR-2110. We conducted luciferase reporter assays to examine whether all three are true target sites of miR-2110. As shown in **Fig 3B**, a fragment of wildtype TSKU 3’UTR (which includes nucleotides 1st– 900th from the 5’ end of the 3’UTR) was cloned into pmiRGlo luciferase reporter plasmid. The three predicted target sites were mutated individually to generate TSKU-mut1, TSKU-mut2 and TSKU-mut3, respectively. A mutant with all three predicted target sites mutated was also generated (TSKU-mut1-3). As shown in **Fig 3C**, in BE(2)-C cells expressing wildtype 3’UTR (TSKU-WT), miR-2110 mimic significantly repressed luciferase activity compared to cells transfected with control oligo. Comparing to control oligo, miR-2110 mimic also significantly repressed luciferase activity in BE(2)-C cells expressing each of the three individually mutated 3’UTRs, whereas mutation all three sites together completely abolished the luciferase repressing effect by miR-2110 mimic. In addition, the magnitude of repression by miR-2110 in cells expressing the individually mutated 3’UTRs was significantly smaller than in cells expressing TSKU-WT, showing significantly higher luciferase activities in cells expressing mutated 3’UTRs relative to cells with TSKU-WT. These results together indicate that all three predicted target sites are true functional target sites of miR-2110 and contribute to the repression of TSKU protein expression by miR-2110; when only one of the target sites is mutated, the other two sites still contribute to the repression of TSKU expression by miR-2110.

In order to further demonstrate the interaction between miR-2110 and *TSKU* 3’UTR, we examined the effect of miR-2110 mimic on cell differentiation in BE(2)-C cells expressing the TSKU-WT luciferase reporter. As shown in **Fig 3D**, the effect of miR-2110 mimic on neurite outgrowth was significantly reduced by TSKU-WT over-expression compared to cells without TSKU-WT expression. These results indicate that the over-expressed 3’UTR of *TSKU* acts as a sponge that binds to and sequesters miR-2110 mimic–this reduces the effective concentration of miR-2110 mimic, reducing the differentiation-inducing effect of miR-2110 mimic. Correspondingly, the inhibition of cell survival by miR-2110 mimic was also significantly diminished by TSKU-WT over-expression **(Fig 3E**).

Altogether, our results demonstrate that miR-2110 represses TSKU protein expression via three target sites in the 3’UTR of *TSKU* mRNA.

### miR-2110 and TSKU has differential effects on cell differentiation and survival in neuroblastoma cell lines with different genetic backgrounds

**Fig 4:**
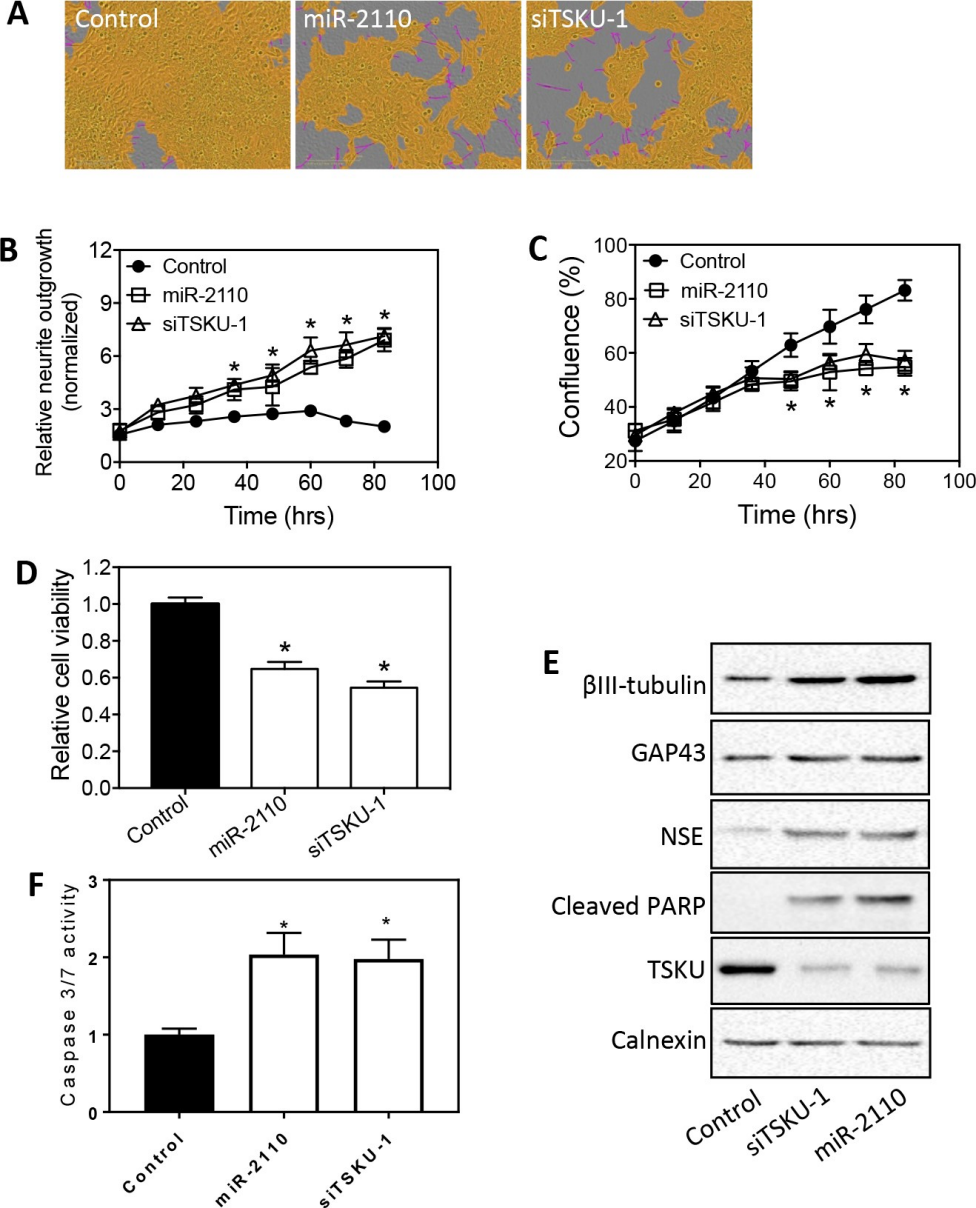
Effect of miR-2110 over-expression and TSKU knockdown on SKNDZ cell differentiation and survival. SKNDZ cells were transfected with control oligo, miR-2110 mimic or siTSKU-1 (25 nM), and neurite outgrowth, cell confluence, cell viability and protein expression levels were measured as above. (**A**) Representative images showing the effect of miR-2110 mimic and siTSKU-1 on neurite outgrowth. (**B-C)** Time-dependent neurite outgrowth (**B**) and cell confluence change (**C**) after each treatment. *, *p* < 0.05 for both miR-2110 and siTSKU-1 compared to the control at the respective time points. (**D**) Cell viability associated with each treatment. *, *p* < 0.05 compared to control. (**E**) Effect of miR-2110 mimic and siTSKU-1 on protein expression of cell differentiation markers (βIII-tubulin, GAP43, NSE), apoptosis marker (cleaved PARP) and TSKU, with calnexin measured as a loading control. (**F)** Effect of siTSKU-1 and miR-2110 mimic on caspase 3/7 activity. *, *p* < 0.05 compared to control.

We next investigated the effect of miR-2110 mimic and siTSKU in a panel of neuroblastoma cell lines with different genetic backgrounds (**S3 Table**). We first examined another cell line known to be sensitive to differentiation induction by RA and differentiation-inducing miRNAs [21]. As shown in **Fig 4A and 4B**, both miR-2110 mimic and siTSKU-1 increased neurite outgrowth in SKNDZ in a time-dependent manner. Correspondingly, miR-2110 mimic and siTSKU-1 also decreased cell proliferation and viability **(Fig 4C and 4D**), and increased expression of molecular differentiation markers (**Fig 4E**). In addition, the magnitude of the effect of TSKU knockdown on neurite outgrowth, cell viability and proliferation were comparable to that of miR-2110 mimic, further supporting TSKU as a key target mediating the effect of miR-2110 in SKNDZ cells. Correspondingly, the apoptotic markers, including increased cleaved PARP protein levels (**Fig 4E**) and caspase 3/7 activity (**Fig 4F**), were also detected following miR-2110 mimic and siTSKUs, consistent with results shown in **Fig 2**.

**Fig 5:**
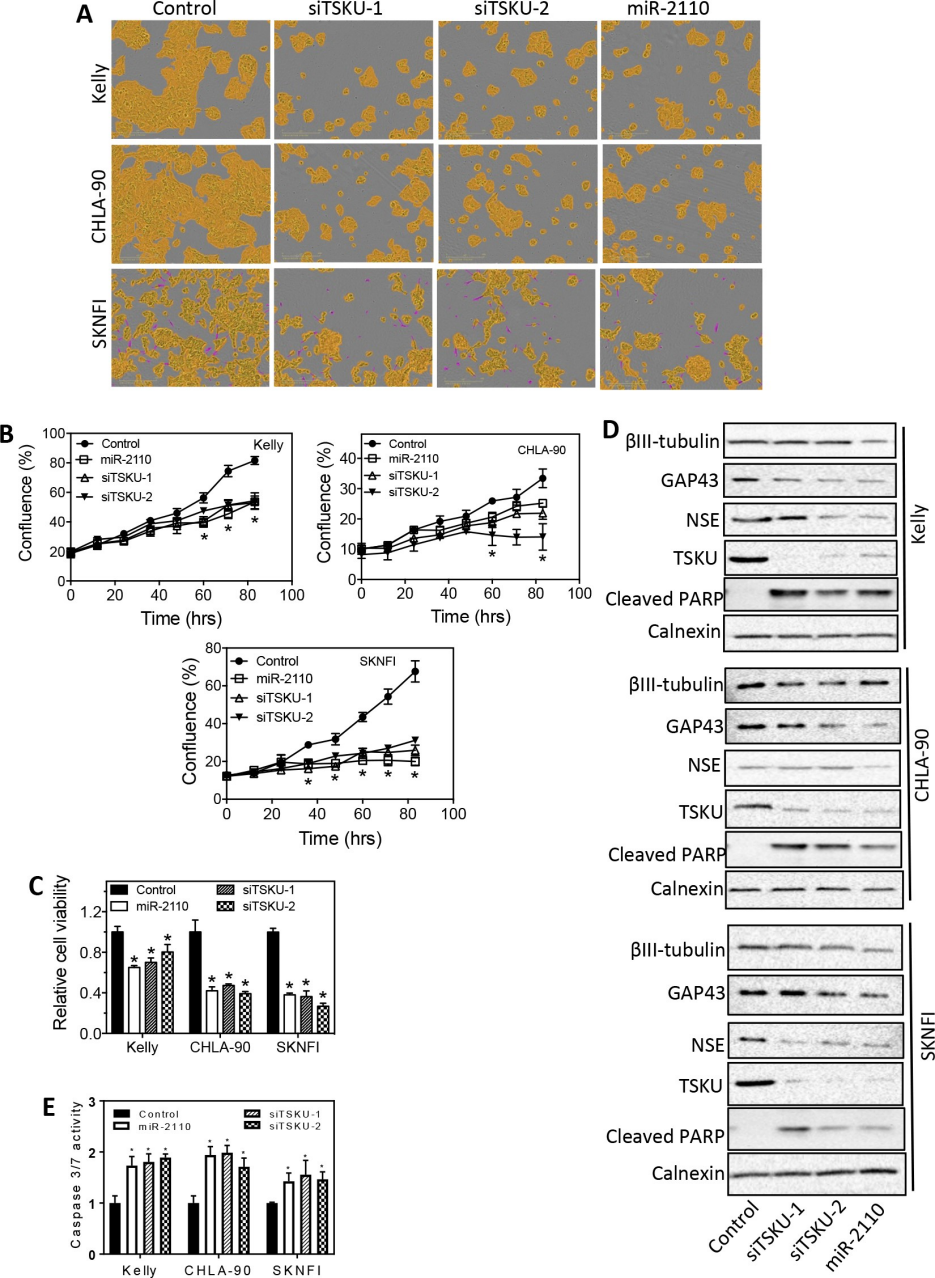
Effect of miR-2110 over-expression and TSKU knockdown on cell differentiation and survival in Kelly, CHLA-90 and SKNFI cells. The indicated cell lines were transfected with as above, and neurite outgrowth, cell confluence, cell viability and protein expression levels were measured as above. **A**, Cell images showing that miR-2110 mimic and siTSKUs did not induce neurite outgrowth in the three cell lines. **B,** Time-dependent cell confluence change after each treatment. *, *p* < 0.05 for all three treatments (miR-2110, siTSKU-1 and siTSKU-2) compared to the control at the respective time points. (**C**) Cell viability associated with each treatment. *, *p* < 0.05 compared to control. (**D**) Effect of miR-2110 mimic and siTSKU-1 on protein expression of cell differentiation markers (βIII-tubulin, GAP43, NSE), apoptosis marker (cleaved PARP) and TSKU, with calnexin measured as a loading control. (**E)** Effect of siTSKUs and miR-2110 mimic on caspase 3/7 activity. *, *p* < 0.05 compared to control.

We next examined three additional neuroblastoma cell lines, Kelly, CHLA-90 and SKNFI, which are relative resistant to differentiation induction by various differentiation-inducing agents [22, 23]. As shown in **Fig 5A,** miR-2110 mimic and siTSKU did not induce neurite outgrowth. However, miR-2110 mimic and siTSKUs significantly decreased cell proliferation and survival in all cell lines (**Fig 5B and 5C**), and miR-2110 mimic and siTSKUs also increased cleaved PARP levels (**Fig 5D**) and caspase 3/7 activity (**Fig 5E)**. **Fig 5D** also shows miR-2110 mimic and siTSKUs did not increase the protein levels of the molecular differentiation markers in these cell lines, further confirming cell differentiation were not induced. These results together indicate that miR-2110 mimic and siTSKUs only reduced cell survival but did not induce cell differentiation in these cell lines. In all cell lines, the extent of TSKU protein knockdown by siTSKU was confirmed by western blot (**Fig 5D**).

Altogether, our investigation indicate that, although neuroblastoma cells with different genetic backgrounds show differential response to miR-2110 mimic and siTSKUs with respect to induced cell differentiation, miR-2110 mimic and siTSKUs have an effective generic effect in reducing neuroblastoma cell survival and proliferation by inducing apoptosis.

### Low tumor miR-2110 levels are significantly correlated with high tumor *TSKU* mRNA levels and poor survival of neuroblastoma patients

**Fig 6:**
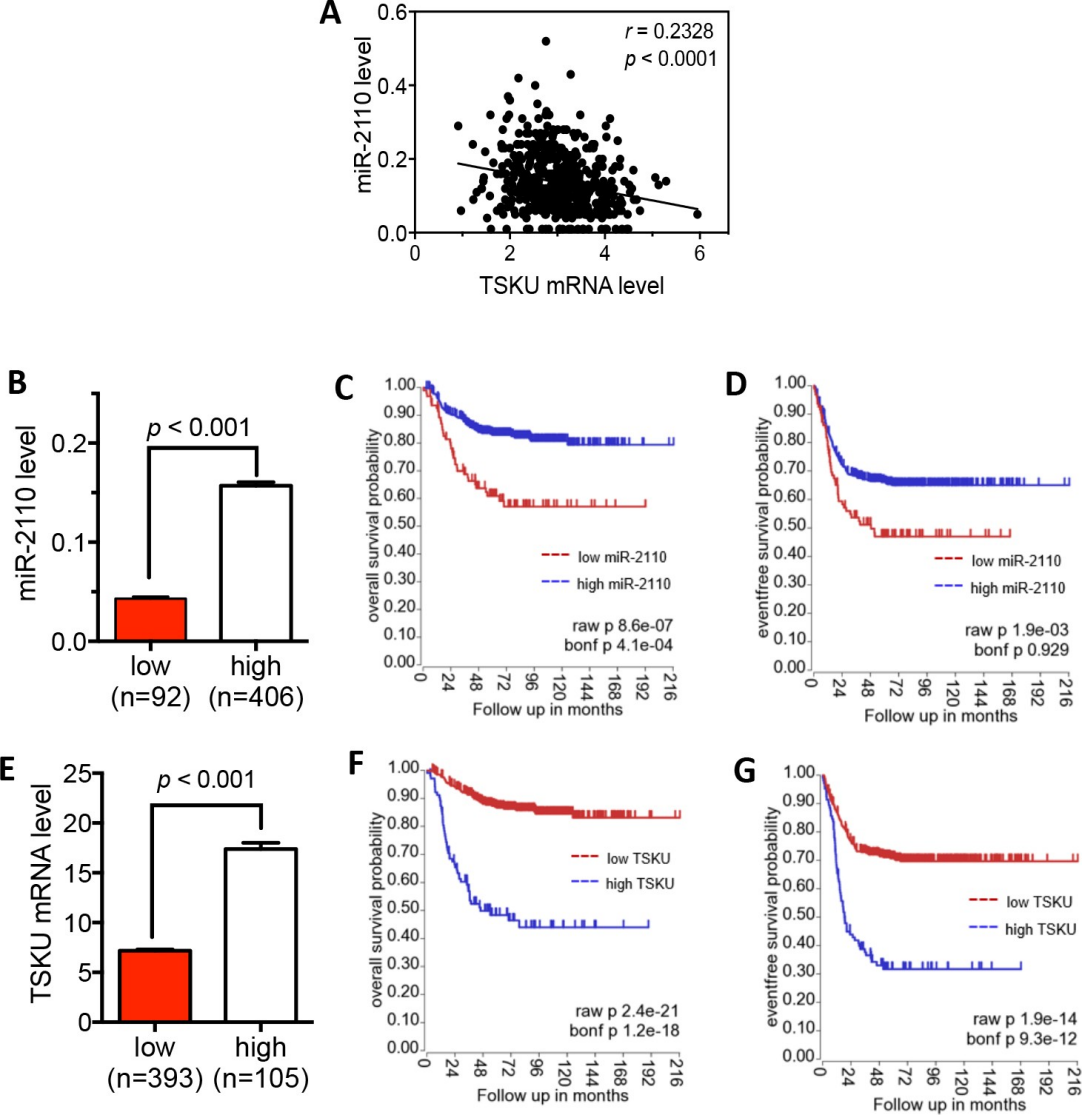
Correlation of tumor miR-2110 and *TSKU* mRNA levels with survival of neuroblastoma patients. (**A**) Correlation between tumor miR-2110 and *TSKU* mRNA levels was assessed using Pearson correlation with *p* < 0.05 considered statistically significant. (**B-G**) Correlation of tumor miR-2110 and *TSKU* mRNA levels with patient survival. The patients were divided into high and low groups based on miR-2110 (**B**) and *TSKU* mRNA (**E**) levels. The Kaplan-Meier overall survival curves and event-free survival curves were plotted for the miR-2110 (**C, D**) and *TSKU* (**F, G**) groups, respectively. Bonf, Bonferroni.

In order to evaluate the clinical relevance of miR-2110-mediated down-regulation of *TSKU* in neuroblastoma patients, we examined tumor miR-2110 and *TSKU* mRNA levels in neuroblastoma patients as well as their correlation with patient survival based on the published SEQC neuroblastoma patient dataset [19, 24]. As shown in **Fig 6A,** there is a significant negative correlation between miR-2110 and *TSKU* mRNA levels (R = 0.23, p < 0.0001). This supports the potential clinical relevance of miR-2110-mediated down-regulation of *TSKU* expression in neuroblastoma tumorigenesis.

To examine the correlation of tumor miR-2110 levels with neuroblastoma patient survival, the patients were classified by tumor miR-2110 levels into high or low groups. As shown in **Fig 6B**, the mean miR-2110 levels are significantly different between the two groups (P < 0.001). Kaplan–Meier survival analysis (**Fig 6C and 6D**) shows that both the overall and event-free survival of patients in the low miR-2110 group is significantly lower than those of patients in the high miR-2110 group based on raw *p* value, although the event-free survival comparison did not reach statistical significance according to the Bonferroni (Bonf) corrected *p* value. We next classified the patients into low and high groups by *TSKU* mRNA expression, as shown in **Fig 6E**. Examination of patient survival in these two groups shows that both the overall and event-free survival of patients in the high *TSKU* group are significantly lower than those of patients in the low *TSKU* group according to both raw and Bonf *p* values (**Fig 6F and 6G**).

**Fig 7:**
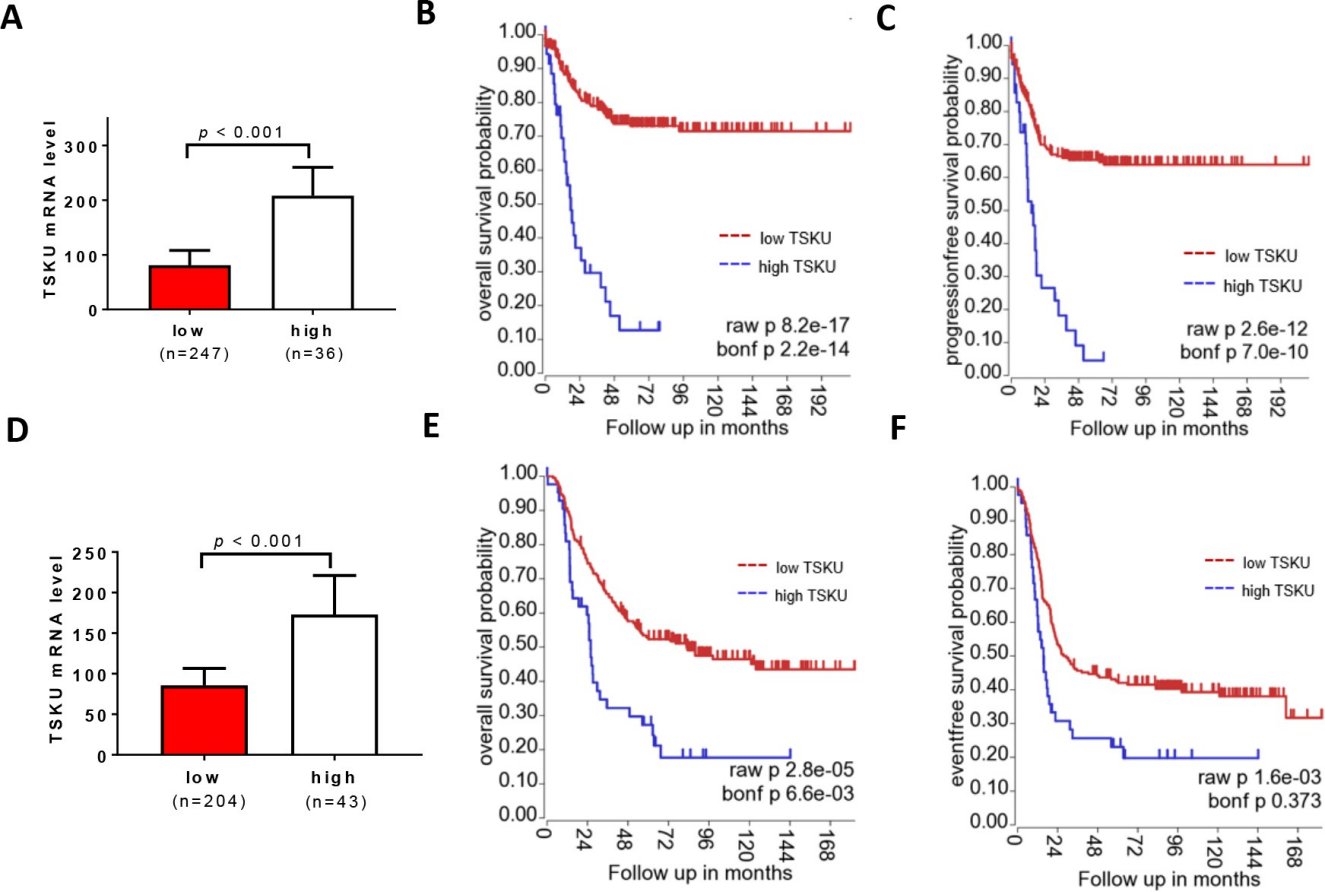
Correlation of TSKU mRNA levels with survival of neuroblastoma patients in the NRC and TARGET dataset. The patients were divided into high and low groups based on *TSKU* mRNA levels from NRC dataset (**A**) and TARGET dataset (**D**). The Kaplan-Meier overall survival curves and event-free survival curves were plotted for the NRC (**B, C**) and TARGET (**E, F**) dataset, respectively.

To further examine the correlation of tumor *TSKU* levels with neuroblastoma patient survival, we analyzed two additional datasets: NRC (**Fig 7A–7C**) and TARGET (**Fig 7D–7F**) datasets [19] (http://r2.amc.nl). As above, the neuroblastoma patients were classified by tumor *TSKU* mRNA levels into high or low groups in the each dataset. As shown in **Fig 7A and 7D**, the mean *TSKU* levels are significantly different between the two groups (*P* < 0.001). In the NRC dataset, Kaplan–Meier survival analysis shows that both the overall survival (**Fig 7B)** and event-free survival (**Fig 7C)** of patients in the low *TSKU* group are significantly lower than those of patients in the high *TSKU* group based on both the raw and Bonf *p* values. In the TARGET dataset, the overall survival (**Fig 7E)** of patients in the low *TSKU* group are significantly lower than those of patients in the high *TSKU* group, although the event-free survival comparison did not reach statistical significance according to the Bonf *p* value.

Unfortunately, the miRNA expression data is not available from these two datasets.

## Discussion

In this study, we demonstrated that miR-2110 functioned as a generic repressor of neuroblastoma cell survival and growth and identified *TSKU* as the direct target of miR-2110 that play the major role in mediating such function. We further found that low tumor miR-2110 levels were correlated with high tumor *TSKU* mRNA levels, and that both low miR-2110 and high *TSKU* mRNA levels were correlated with poor neuroblastoma patient survival, suggesting that the miR-2110-mediated down-regulation of *TSKU* expression is an important determinant of neuroblastoma prognosis.

miR-2110 has been related to tumorigenesis in previous studies. Zhu, *et al*. first discovered human miR-2110 when investigating miRNAs expressed in nasopharyngeal carcinomas (NPC) and normal tissues [25]. However, since its expression in both NPC and normal tissues were low [25], miR-2110 was not found to be differentially expressed between the two. A later study by Gaedcke, *et al*. showed that miR-2110 was down-regulated in rectal cancer relative to normal adjacent tissue [26]. More recently, Ferracin, *et al*. found that plasma levels of miR-2110 from colorectal cancer patients were significantly lower than those from normal healthy subjects [27]. These two studies suggest that expression of miR-2110 in cancer cells was suppressed. In this study, we found that low tumor miR-2110 levels were correlated with poor survival neuroblastoma patients, supporting the onco-suppressive function of miR-2110 in neuroblastoma and consistent with previous findings in other cancer types [26, 27].

The miR-2110 target gene *TSKU* encodes a leucine-rich protein belonging to the large family of secreted leucine-rich proteoglycans (SLRPs) [28]. SLRPs have been found to have diverse molecular and cellular functions [29], including regulation of cell survival, proliferation and migration [29–31], and these functions are mediated by diverse molecular mechanisms, including modulation of receptor tyrosine kinase-mediated signaling pathways, activation of Toll-like receptors and TGFR pathways [28, 29]. Multiple SLRPs have been shown to play roles in tumorigenesis [32], and several SLRP members have been shown to function as cell type-specific growth promoters or growth inhibitors. For example, SLRP decorin has been found to have anti-apoptotic effects in epithelial, endothelial and bone marrow stromal cells [33, 34]. However, other studies support the opposite function of decorin with its over-expression inhibiting squamous carcinoma cancer cell proliferation [35–37]. Similarly, another SLRP protein, biglycan, was found to inhibit pancreatic cancer cell proliferation [38], but to favor vascular smooth muscle cell proliferation and migration [39]. In our study, we found that depletion of TSKU in neuroblastoma cells induced cell differentiation and reduced cell viability, supporting its potential oncogenic function. The function of TSKU in cancers has not been exploited prior to our study. Whether such functions of TSKU is also cell-type specific and only limited to neuroblastoma certainly warrants further investigation.

Many studies have shown that SLRPs, as secreted proteins, regulate cell growth by modulating the interactions of cells with extracellular matrix. Little attention has been given to their functions as independent intracellular functions [29]. Our results show that the intracellular TSKU protein can be detected by Western blot, and that its intracellular protein levels are correlated with its function in regulating cell survival and differentiation. These results strongly suggest that TSKU has independent functions as an intracellular protein. However, it is unclear whether the detected intracellular TSKU protein is the endogenous product of the cells or is internalized from the extracellular environment. This certainly warrants further investigation in the future.

The role of TSKU in regulating cell differentiation has been previously suggested in several cell types including neuronal cells [40–42]. For example, Hossain *et al*. showed that TSKU overexpression inhibited anterior olfactory neural and cortical neurite outgrowth [40]. Niimori *et al*. found that TSKU treatment inhibited myofibroblast differentiation from NIH3T3 cells [41]. In another study, Niimori et *al*. demonstrated that knockout of TSKU led to a delay of the hair cycle, which suggests that TSKU plays a role in maintenance of hair follicular stem cells [42]. The above findings are consistent with our finding that knockdown of TSKU expression induces cell differentiation in certain neuroblastoma cell lines. An interesting observation in our study is that neuroblastoma cells with different genetic backgrounds show distinct responses to miR-2110-induced cell differentiation, with miR-2110 effectively inducing differentiation in RA-sensitive cell lines but not in RA-resistant cell lines. Given the cell type-specific functions observed in other SLRP family members [33–39], it is not surprising that the function of TSKU in regulating neuroblastoma cell differentiation is also cell context-specific, with TSKU knockdown, either by miR-2110 mimic or siTSKUs, inducing cell differentiation traits in some cell lines but not in the others. Previous studies have shown that cell lines Kelly, CHLA-90 and SKNFI could be differentiated by other differentiation agents, including differentiation-inducing miRNAs [12], indicating that the lack of response of these cells to miR-2110-induced differentiation is not due to complete loss of the differentiation machinery in these cells. Our results support the notion that multiple non-overlapping pathways exist for directing neuroblastoma cell differentiation, and each specific neuroblastoma cell line may express only one or more of the differentiation pathways but lack the others, as determined by the intrinsic genetic traits of the cell lines. This explains why other miRNAs such as miR-506-3p effectively induced differentiation in the cell lines Kelly, CHLA-90 and SKNFI [12], whereas miR-2110 did not. The differential responses of neuroblastoma cell lines to differentiation-inducing agents also highlight the importance of personalized therapy when using differentiation agents. miR-2110 certainly needs to be further investigated in a larger panel of neuroblastoma cell lines in order to identify the specific genetic traits that are correlated with the response to miR-2110; the molecular mechanisms underlying such differential responses also warrant further investigation.

In summary, our study provides the first comprehensive characterization of the potential tumor-suppressive function of miR-2110. Our findings provide the experimental and retrospective clinical evidence to support the development of new prognosis markers and therapeutic approaches through the miR-2110-TSKU axis. The differential response of neuroblastoma cells with different genetic backgrounds to the manipulation of miR-2110 and *TSKU* expression certainly needs to be further investigated for further evaluating their prognostic and therapeutic values in different genetic subgroups of neuroblastoma.

## Supporting information

S1 Table Effect of miR-2110 target gene knockdown on neurite outgrowth of BE(2)-C cells. Cells were transfected with the indicated siRNAs and neurite outgrowth was measured as above. Shown are (1) the siRNA name, (2) the mean value of normalized neurite length from three independent experiments, (3) the standard deviation (SD) of normalized neurite length from the three experiments, (4) *p* value, (5) *q* value and (6) neurite outgrowth discovery. *, three different siRNAs were pooled. **, Yes, discovered as significantly inducing neurite outgrowth based on *p* < 0.05 and FDR (*q* value) < 0.2; No, not discovered as significantly inducing neurite outgrowth based on *p* < 0.05 and FDR (*q* value) < 0.2.
(DOCX)

S2 Table Effect of miR-2110 target gene knockdown on survival of BE(2)-C cells. Cells were treated as described in S1 Table legend. Shown are (1) the siRNA name, (2) the mean value of normalized cell viability from three independent experiments, (3) the SD of cell viability, (4) *p* value, (5) *q* value and (6) cytotoxicity discovery. *, Three different siRNAs were pooled. **, Yes, discovered as significantly decreasing cell viability based on *p* < 0.05 and FDR (*q* value) < 0.2; No, not discovered as significantly decreasing cell viability based on *p* < 0.05 and FDR (*q* value) < 0.2.
(DOCX)

S3 Table Genetic backgrounds of neuroblastoma cell lines used in this study. Shown are the name of the cell line, age and gender of the patient, stage of the tumor from which the cell line was derived, chromosome 1p and 17 alterations, and MYCN gene amplification status. unk, unknown; Chr, Chromosome; ampl, amplification.
(DOCX)

S4 Table Gene expression array data associated with miR-2110 mimic treatment in BE(2)-C cells. Cells were treated with or without 25 nM of miR-2110 mimic (miR-2110 mimic and mock, respectively, as shown in the Table) for 24 hours. mRNA was isolated and mRNA expression array were performed as described in **Materials and Methods**.
(XLS)
