# Supplementary material for: microRNA-2110 functions as an onco-suppressor in neuroblastoma by directly targeting Tsukushi
Source: PLoS One. 2018 Dec 14;13(12):e0208777. doi: 10.1371/journal.pone.0208777 (PMC6294380; doi:10.1371/journal.pone.0208777)
Supplement: S1 Table — Cells were transfected with the indicated siRNAs and neurite outgrowth was measured as above. Shown are (1) the siRNA name, (2) the mean value of normalized neurite length from three independent experiments, (3) the standard deviation (SD) of normalized neurite length from the three experiments, (4) p value, (5) q value and (6) neurite outgrowth discovery. *, three different siRNAs were pooled. **, Yes, discovered as significantly inducing neurite outgrowth based on p < 0.05 and FDR (q value) < 0.2; No, not discovered as significantly inducing neurite outgrowth based on p < 0.05 and FDR (q value) < 0.2. (DOCX) [file pone.0208777.s001.docx]

| **(1) siRNAs** | **(2) Mean** | **(3) SD** | **(4) *p*** | **(5) *q*** | **(6) Discovery**** |
| --- | --- | --- | --- | --- | --- |
| siTSKU-1 | 2.207 | 0.424 | 1.9E-13 | 0.000 | Yes |
| siTSKU-2 | 2.083 | 0.281 | 2.0E-11 | 0.000 | Yes |
| siE2F2* | 1.657 | 0.208 | 1.8E-05 | 0.000 | Yes |
| siFOXM1* | 1.362 | 0.249 | 0.014 | 0.061 | Yes |
| siNOP56-1 | 1.334 | 0.449 | 0.022 | 0.089 | Yes |
| siPATL1-1 | 1.215 | 0.194 | 0.128 | 0.314 | No |
| siELK1-2 | 1.207 | 0.338 | 0.141 | 0.314 | No |
| siELK1-1 | 1.164 | 0.320 | 0.233 | 0.491 | No |
| siNOP56-2 | 1.146 | 0.334 | 0.282 | 0.565 | No |
| siSORBS3-1 | 1.137 | 0.131 | 0.312 | 0.593 | No |
| siSLC35E1-2 | 1.103 | 0.158 | 0.429 | 0.652 | No |
| siDDN-2 | 1.079 | 0.219 | 0.524 | 0.676 | No |
| siSLC38A1-2 | 1.068 | 0.182 | 0.574 | 0.696 | No |
| siSYN1-1 | 1.050 | 0.254 | 0.657 | 0.751 | No |
| siSLC25A23-1 | 1.033 | 0.276 | 0.736 | 0.818 | No |
| siOAF-1 | 1.005 | 0.213 | 0.878 | 0.900 | No |
| siPATL1-2 | 0.999 | 0.183 | 0.912 | 0.912 | No |
| siSYN1-2 | 0.945 | 0.259 | 0.808 | 0.850 | No |
| siSLC25A23-2 | 0.943 | 0.337 | 0.801 | 0.850 | No |
| siSLC35E1-1 | 0.914 | 0.313 | 0.657 | 0.751 | No |
| siDDN-1 | 0.893 | 0.063 | 0.562 | 0.696 | No |
| siTTL-2 | 0.882 | 0.356 | 0.515 | 0.676 | No |
| siMARCKSL1-2 | 0.875 | 0.116 | 0.484 | 0.668 | No |
| siSORBS3-2 | 0.868 | 0.245 | 0.456 | 0.652 | No |
| siRALY-1 | 0.866 | 0.234 | 0.450 | 0.652 | No |
| siG3BP1-2 | 0.855 | 0.270 | 0.408 | 0.652 | No |
| siOAF-2 | 0.844 | 0.076 | 0.368 | 0.614 | No |
| siTMEM69-2 | 0.842 | 0.146 | 0.360 | 0.614 | No |
| siSTRN4-2 | 0.838 | 0.037 | 0.347 | 0.614 | No |
| siUSP13-1 | 0.756 | 0.345 | 0.140 | 0.314 | No |
| siUSP13-2 | 0.751 | 0.208 | 0.133 | 0.314 | No |
| siSTRN4-1 | 0.727 | 0.144 | 0.097 | 0.276 | No |
| siPMM2-1 | 0.690 | 0.188 | 0.057 | 0.176 | No |
| siG3BP1-2 | 0.666 | 0.167 | 0.040 | 0.134 | No |
| siMARCKSL1-1 | 0.657 | 0.346 | 0.035 | 0.127 | No |
| siTMEM69-1 | 0.600 | 0.225 | 0.013 | 0.061 | No |
| siRALY-2 | 0.584 | 0.235 | 0.010 | 0.057 | No |
| siPMM2-2 | 0.563 | 0.482 | 0.007 | 0.045 | No |
| siTTL-1 | 0.463 | 0.372 | 0.001 | 0.007 | No |
| siSLC38A1-1 | 0.401 | 0.065 | 2.0E-04 | 0.002 | No |
