# Supplementary material for: microRNA-2110 functions as an onco-suppressor in neuroblastoma by directly targeting Tsukushi
Source: PLoS One. 2018 Dec 14;13(12):e0208777. doi: 10.1371/journal.pone.0208777 (PMC6294380; doi:10.1371/journal.pone.0208777)
Supplement: S2 Table — Cells were treated as described in S1 Table legend. Shown are (1) the siRNA name, (2) the mean value of normalized cell viability from three independent experiments, (3) the SD of cell viability, (4) p value, (5) q value and (6) cytotoxicity discovery. *, Three different siRNAs were pooled. **, Yes, discovered as significantly decreasing cell viability based on p < 0.05 and FDR (q value) < 0.2; No, not discovered as significantly decreasing cell viability based on p < 0.05 and FDR (q value) < 0.2. (DOCX) [file pone.0208777.s002.docx]

| **(1) siRNA** | **(2) Mean** | **(3) SD** | **(4) *p*** | **(5) *q*** | **(6) Discovery**** |
| --- | --- | --- | --- | --- | --- |
| siTSKU-1 | 0.559 | 0.286 | 2.1E-04 | 0.004 | Yes |
| siMARCKSL1-2 | 0.561 | 0.253 | 2.3E-04 | 0.004 | Yes |
| siSLC38A1-2 | 0.648 | 0.357 | 0.003 | 0.036 | Yes |
| siOAF-2 | 0.693 | 0.292 | 0.009 | 0.085 | Yes |
| siTMEM69-2 | 0.703 | 0.198 | 0.012 | 0.087 | Yes |
| siTSKU-2 | 0.704 | 0.094 | 0.024 | 0.112 | Yes |
| siSLC25A23-2 | 0.782 | 0.065 | 0.063 | 0.193 | No |
| siNOP56-2 | 0.786 | 0.333 | 0.068 | 0.193 | No |
| siELK1-2 | 0.813 | 0.349 | 0.110 | 0.242 | No |
| siOAF-1 | 0.866 | 0.141 | 0.251 | 0.389 | No |
| siG3BP1-2 | 0.875 | 0.246 | 0.284 | 0.423 | No |
| siSLC25A23-1 | 0.882 | 0.128 | 0.314 | 0.434 | No |
| siNOP56-1 | 0.882 | 0.182 | 0.315 | 0.434 | No |
| siTTL-2 | 0.939 | 0.072 | 0.604 | 0.702 | No |
| siSORBS3-2 | 0.951 | 0.132 | 0.680 | 0.744 | No |
| siRALY-2 | 0.960 | 0.118 | 0.737 | 0.762 | No |
| siSTRN4-2 | 0.980 | 0.202 | 0.868 | 0.828 | No |
| siPMM2-2 | 1.011 | 0.154 | 0.921 | 0.856 | No |
| siPATL1-2 | 1.019 | 0.040 | 0.864 | 0.828 | No |
| siELK1-1 | 1.030 | 0.151 | 0.795 | 0.799 | No |
| siSTRN4-1 | 1.041 | 0.193 | 0.721 | 0.762 | No |
| siG3BP1-1 | 1.053 | 0.186 | 0.643 | 0.725 | No |
| siPATL1-1 | 1.066 | 0.075 | 0.565 | 0.678 | No |
| siRALY-1 | 1.081 | 0.035 | 0.485 | 0.601 | No |
| siDDN-2 | 1.088 | 0.163 | 0.447 | 0.573 | No |
| siUSP13-2 | 1.100 | 0.105 | 0.388 | 0.515 | No |
| siSYN1-2 | 1.141 | 0.154 | 0.226 | 0.365 | No |
| siSLC35E1-2 | 1.147 | 0.120 | 0.206 | 0.348 | No |
| siSLC38A1-1 | 1.158 | 0.188 | 0.174 | 0.308 | No |
| siMARCKSL1-1 | 1.163 | 0.264 | 0.161 | 0.299 | No |
| siSYN1-1 | 1.179 | 0.059 | 0.125 | 0.244 | No |
| siE2F2* | 1.182 | 0.166 | 0.117 | 0.242 | No |
| siTTL-1 | 1.183 | 0.195 | 0.116 | 0.242 | No |
| siSLC35E1-1 | 1.185 | 0.122 | 0.113 | 0.242 | No |
| siDDN-1 | 1.201 | 0.259 | 0.085 | 0.226 | No |
| siUSP13-1 | 1.228 | 0.221 | 0.051 | 0.172 | No |
| siPMM2-1 | 1.229 | 0.126 | 0.050 | 0.172 | No |
| siSORBS3-1 | 1.258 | 0.286 | 0.027 | 0.112 | No |
| siTMEM69-1 | 1.273 | 0.393 | 0.020 | 0.105 | No |
| siFOXM1* | 1.278 | 0.046 | 0.018 | 0.105 | No |
