# Supplementary material for: microRNA-2110 functions as an onco-suppressor in neuroblastoma by directly targeting Tsukushi
Source: PLoS One. 2018 Dec 14;13(12):e0208777. doi: 10.1371/journal.pone.0208777 (PMC6294380; doi:10.1371/journal.pone.0208777)
Supplement: S3 Table — Shown are the name of the cell line, age and gender of the patient, stage of the tumor from which the cell line was derived, chromosome 1p and 17 alterations, and MYCN gene amplification status. unk, unknown; Chr, Chromosome; ampl, amplification. (DOCX) [file pone.0208777.s003.docx]

| **Cell line** | **Age** | **Gender** | **Stage** | **Chr 1p alteration** | **Chr 17 alteration** | **MYCN ampl.** |
| --- | --- | --- | --- | --- | --- | --- |
| BE(2)-C | 2 | male | 4 | Y | N | Y |
| SKNDZ | 2 | female | 4 | Y | Y | N |
| Kelly | unk | unk | unk | unk | unk | Y |
| CHLA-90 | 8.5 | male | 4 | unk | unk | N |
| SKNFI | 11 | male | unk | unk | unk | N |
